# ATF2 knockdown reinforces oxidative stress-induced apoptosis in TE7 cancer cells

**DOI:** 10.1111/jcmm.12071

**Published:** 2013-06-25

**Authors:** Diana Walluscheck, Angela Poehlmann, Roland Hartig, Uwe Lendeckel, Peter Schönfeld, Agnes Hotz-Wagenblatt, Kathrin Reissig, Khuloud Bajbouj, Albert Roessner, Regine Schneider-Stock

**Affiliations:** a Department of Pathology, Otto-von-Guericke UniversityMagdeburg, Germany; b Department of Molecular and Clinical Immunology, Otto-von-Guericke UniversityMagdeburg, Germany; c Department of Experimental Internal Medicine, Otto-von-Guericke UniversityMagdeburg, Germany; d Institute of Medical Biochemistry and Molecular Biology University Medicine, Ernst-Moritz-Arndt-UniversityGreifswald, Germany; e Department of Biochemistry and Cell Biology, Otto-von-Guericke UniversityMagdeburg, Germany; f Core Facility Genomics Proteomics, German Cancer Research Center (DKFZ)Heidelberg, Germany; g University of Hawaii Cancer Center, University of HawaiiHonolulu, HI, USA; h Experimental Tumor Pathology Department of Pathology, University of ErlangenErlangen, Germany

**Keywords:** oxidative stress-induced DNA damage, cell cycle arrest, ATF2 knockdown, increase in apoptosis sensitivities, combined treatment, p21^WAF1^

## Abstract

Cancer cells showing low apoptotic effects following oxidative stress-induced DNA damage are mainly affected by growth arrest. Thus, recent studies focus on improving anti-cancer therapies by increasing apoptosis sensitivity. We aimed at identifying a universal molecule as potential target to enhance oxidative stress-based anti-cancer therapy through a switch from cell cycle arrest to apoptosis. A cDNA microarray was performed with hydrogen peroxide-treated oesophageal squamous epithelial cancer cells TE7. This cell line showed checkpoint activation *via* p21^WAF1^, but low apoptotic response following DNA damage. The potential target molecule was chosen depended on the following demands: it should regulate DNA damage response, cell cycle and apoptosis. As the transcription factor ATF2 is implicated in all these processes, we focused on this protein. We investigated checkpoint activation *via* ATF2. Indeed, ATF2 knockdown revealed ATF2-triggered p21^WAF1^ protein expression, suggesting p21^WAF1^ transactivation through ATF2. Using chromatin immunoprecipitation (ChIP), we identified a hitherto unknown ATF2-binding sequence in the p21^WAF1^ promoter. p-ATF2 was found to interact with p-c-Jun, creating the AP-1 complex. Moreover, ATF2 knockdown led to c-Jun downregulation. This suggests ATF2-driven induction of c-Jun expression, thereby enhancing ATF2 transcriptional activity *via* c-Jun-ATF2 heterodimerization. Notably, downregulation of ATF2 caused a switch from cell cycle arrest to reinforced apoptosis, presumably *via* p21^WAF1^ downregulation, confirming the importance of ATF2 in the establishment of cell cycle arrest. 1-Chloro-2,4-dinitrobenzene also led to ATF2-dependent G2/M arrest, suggesting that this is a general feature induced by oxidative stress. As ATF2 knockdown also increased apoptosis, we propose ATF2 as a target for combined oxidative stress-based anti-cancer therapies.

## Introduction

Conventional non-surgical anti-cancer therapies, such as radio- and chemotherapy, often poorly induce apoptosis in tumour cells [1]. It is now well-established that reactive oxygen species (ROS) can trigger apoptotic cell death. Frequently applied chemotherapeutics, e.g. 5-Fluorouracil and Ruthenium-based drugs, as well as treatment by radiation, can act through oxidative stress induction ([2, 3], reviewed in [4]). Thus, the current tasks of scientists are (*i* ) to better understand the molecular responses of tumours to oxidative stress for predicting the complete pathological response, and (*ii* ) to develop or improve therapeutic concepts. In this context, oesophagus cancer, which is highly malignant and resistant to apoptosis, is the subject of research [5–7]. As the squamous oesophageal cancer cell line TE7 with dysregulated p53 shows only poor apoptotic outcome to oxidative stress, it is an appropriate model for this disease [8]. In addition, oxidative damage seems to play a role in the pathogenesis of oesophageal cancer [9].

Some studies focus on mimicking oxidative stress-based anti-cancer therapies either by inducing ROS production or diminishing the capacity of the endogenous anti-oxidant defence system [10]. The response of cells to oxidative damage involves multiple mechanisms including the activation of redox-sensitive signal transduction cascades, culminating in transcription factors activation, and the subsequent induction of their target genes. These pathways play a role in DNA repair, cell cycle arrest and apoptosis.

To improve therapeutic outcome, targeting of important DNA damage checkpoint proteins, which may affect cell cycle regulation, has increasingly been considered as a promising strategy that switches growth inhibition to desired apoptotic response. Target proteins include serine/threonine protein kinases, such as Ataxia telangiectasia mutated (ATM), ataxia telangiectasia and Rad3-related protein (ATR), extracellular signal-regulated kinases (ERK), p38 mitogen-activated protein kinases (p38), c-Jun *N*-terminal kinases (JNK) and, especially, the checkpoint kinase 1 (Chk1) [11–17]. The identification of novel potential targets that are key proteins in cell cycle regulation is increasingly becoming a focus of research.

Growth inhibition is triggered by the mitogen-activated protein kinase (MAPK) pathway (reviewed in [17]), which activates the activating transcription factor 2 (ATF2) and jun proto-oncogene (c-Jun) [18]. Both transcription factors regulate target genes, such as cyclin-dependent kinase inhibitor 1A (CDKN1A) [19], encoding the protein p21^WAF1^, thereby inducing cell cycle arrest (reviewed in [20]). To transactivate target genes, ATF2 and c-Jun build a complex referred to as activator protein 1 (AP-1). In addition, ATF2 can also function after homodimerization [21]. ATF2 is an important DNA damage response protein with subsequent regulation of cell cycle progression [22]. Therefore, it has the potential to be a target to switch cancer cells to apoptosis. Accordingly, Abbas *et al*. repressed melanoma cell growth by inhibiting the ATF2 protein [23].

In this study, we were interested in identifying a key protein acting in cell cycle regulation, which is able to reinforce apoptosis following its inhibition. This key protein should be involved in DNA damage response. Hydrogen peroxide (H_2_O_2_) induced DNA damage, cell cycle arrest and minor apoptosis in TE7 cells. ATF2 was found as a promising target candidate. Indeed, ATF2 partially mediated the G2/M arrest through p21^WAF1^ induction, and we identified a novel ATF2-binding site in the p21^WAF1^ promoter. ATF2 knockdown led to elevated apoptosis. Oxidative stress generated by 1-Chloro-2,4-dinitrobenzene (CDNB) also caused ATF2 activation that mediated the G2/M arrest. Moreover, ATF2 knockdown reinforced apoptosis. These observations taken together suggest the identification of a general mechanism, whereas application of the combined treatment of oxidative stress with knockdown of ATF2 may reinforce the apoptosis rate, which could be of therapeutical interest.

## Materials and methods

### Cell culture, derivation of stable cell lines and treatments

Human cell line TE7 derived from an oesophageal squamous cell carcinoma [24], kindly provided by Professor Wael El-Rifai (Vanderbilt University Medical Center, Nashville, USA), was maintained in DMEM with 10% foetal bovine serum (FBS), penicillin (100 U/ml) and streptomycin (100 μg/ml), and cultured in a humidified 5% CO_2_ atmosphere at 37°C.

The cell culture model is based on the treatment of cells with 250 μM H_2_O_2_ or 10 μM CDNB in cell culture medium. In detail, cells were seeded to 50% confluence, cultured for 24 hrs, treated and collected after 0.25, 0.5, 1, 3, 6, 12 and 24 hrs following treatment. To prove upstream JNK signalling, cells were treated with 10 μM of the JNK inhibitor SP600125 1 hr before H_2_O_2_ treatment. All treatments were performed in triplicate.

### Cell death measurement

To detect apoptosis and necrosis, the Annexin-V-FLUOS kit (Roche Diagnostics GmbH, Mannheim, Germany) was used as described previously [25]. Annexin-V binding by phosphatidylserine (PS)-exposing cells was defined as apoptosis, whereas cells stained with both propidiumioide (PI) and Annexin-V or with PI alone were considered as necrotic cells. The experiments were performed in triplicate.

### Comet assay

The CometAssay™ from TREVIGEN, Inc. (Gaithersburg, MD, USA) was performed in triplicate according to the protocol of the supplier with modifications described in the supplementary material of Poehlmann *et al*. [26]. Evaluation was also performed according to their protocol.

### Flow cytometric analysis of DNA content

Harvesting of the cells, collection of supernatants, preparation of samples and staining of the DNA were performed in triplicate as described by Poehlmann *et al*. [26]. The percentage of cells in the appropriate phases of the cell cycle was determined and allocated according to their protocol.

### Cell proliferation ELISA

Cell proliferation was assessed by colorimetric 5-bromo-2′-deoxyuridine (BrdU) immunoassay according to the manufacturer's recommendations (Roche Diagnostics GmbH). Experiments were performed in triplicate.

### Fluorescence immunolabelling analysis

TE7 cells were fixed and permeabilized as described previously [27]. Overnight staining at 4°C was performed with 1:200 anti-*γ*-H2AX (Millipore, Billerica, MA, USA) and 1:400 anti-ATF2 (Cell Signaling Technology Inc., Danvers, MA, USA), followed by an appropriate secondary antibody incubation with 1:100 Fluorescein anti-Rabbit IgG (Vector Laboratories Inc., Burlingame, CA, USA), as well as with 1:400 Cy™3-conjugated AffiniPure Goat Antimouse IgG (Jackson ImmunoResearch Laboratories Inc., Suffolk, UK) for 1 hr at 37°C. Fluorescence immunolabelling analysis was performed in triplicate.

### Small interfering RNA transfection

Target-specific 20-25 nucleotides ATF2 siRNAs (sc-29205, Santa Cruz Biotechnology Inc., Santa Cruz, CA, USA) were used to degrade ATF2 mRNAs and to reduce the amounts of the appropriate protein. We followed the siRNA transfection protocol of the manufacturer (Santa Cruz) with only minor modifications. Cells were treated for 7 hrs with the siRNA transfection mixture composed of siRNAs, transfection reagent (TFR) and transfection medium. The optimal amount of siRNAs was determined as 8 μl diluted in 6 μl TFR, reaching a 60% ATF2 protein knockdown. The transfection mixture was left on the cells, while applying H_2_O_2_ or CDNB. ATF2 knockdown was repeated at least two times.

### cDNA array analysis

To detect mRNA expression of different key genes involved in several pathways following oxidative stress, we performed the GEArray Q Series ‘Human Signal Transduction PathwayFinder Gene Array’ of SuperArray Bioscience Corporation (Frederick, MD, USA; this company has ceased to exist) according to the protocol of the supplier. The representative key genes participate in different signalling pathways, especially in those involved in cell cycle control, DNA damage and apoptosis. Chemiluminescent detection through Syngene Bio Imaging System was carried out three times as described by Schneider-Stock *et al*. [28]. More details are given in the Supporting Information.

### Immunoblotting analysis

Immunoblotting was performed in triplicate as described previously [25]. Immunodetection was carried out with anti-ATF2, -caspase 3, 8, 9, -c-Jun, -cyclin D1, -Bcl-2, -H3, -H2AX, -p-ATF2^Thr69/71^, -p-H3^Ser10^, -p-c-Jun^Ser73^ (Cell Signaling Technology Inc.), -Bax (DakoCytomation Inc., Carpinteria, CA, USA), -*β*-actin (Sigma-Aldrich, St. Louis, MO, USA), -Chk1 (Santa Cruz Biotechnology Inc.), -p300/CBP (Acris Antibodies GmbH, Herford, Germany), -*γ*-H2AX (p-H2AX^Ser139^; Millipore), -p-Chk1^Ser317^ (Novus Biologicals Inc., Littleton, CO, USA), -p21^WAF1^ (Merck, Darmstadt, Germany) and secondary antibodies (anti-mouse and anti-rabbit IgG peroxidase conjugated, Pierce, Rockford, IL, USA). All data from immunoblotting, shown in the figures, are representative of three independent experiments. Fold expression changes were calculated through the ratio of the respective protein expression and *β*-actin. In cases in which the ratio of the controls is zero, the first apparent signal was defined as one.

### Co-immunoprecipitation

Co-immunoprecipitation was performed in triplicate according to ‘Universal Magnetic Co-IP Kits (Version A)’ manual of the company Active Motif (Rixensart, Belgium). Details are given in the Supporting Information.

### Bioinformatic databases

To determine ATF2-binding sequences within the p21^WAF1^ promoter, the genomic sequence of the CDKN1A (NT_007592) promoter region was analysed with the database TFSEARCH: Searching transcription factor binding sites version 1.3 (http://molsun1.cbrc.aist.go.jp/research/db/TFSEARCH.html). CRE-BP, CREB, AP-1 and CRE-BP/CREB sequences were included in the search. Details are given in the Supporting Information.

### ChIP assay

ChIP assay was performed in triplicate according to the manufacturer's protocol of the ChIP-IT™ Express Magnetic Chromatin Immunoprecipitation Kit (Active Motif) with minor modifications, which are given in the Supporting Information.

## Results

### H_2_O_2_ induces DNA damage

**Fig. 1:**
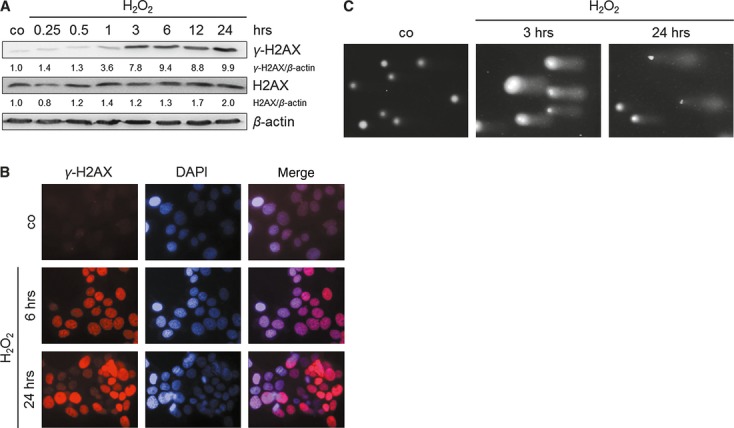
H_2_O_2_ induces DNA damage in the cell line TE7. Cells were treated with 250 μM H_2_O_2_. (**A**) H_2_O_2_ causes accumulation of the DNA damage sensor *γ*-H2AX. Cells were treated and grown for 0.25, 0.5, 1, 3, 6, 12 and 24 hrs. Levels of total H2AX and *γ*-H2AX were determined by immunoblotting using cell lysates. *β*-actin was used as a loading control. Fold expression changes are given below the blots. (**B**) H_2_O_2_ induces *γ*-H2AX foci formation in TE7 nuclei. Representative images of *γ*-H2AX immunostaining of H_2_O_2_-treated cells after 6 and 24 hrs are shown. Nuclear DNA was visualized by 4′,6-diamidino-2-phenylindole (DAPI) counterstaining. (**C**) Comet assay analysis of nuclear DNA revealed DNA damage in TE7 cells as shown by comet tails 3 and 24 hrs following H_2_O_2_ exposure.

The strong oxidant H_2_O_2_ was used to mimic ROS-induced, DNA-damaging anti-cancer therapies as described previously [25, 26]. Initially, molecular events of H_2_O_2_-related oxidative DNA damage were analysed. TE7 cells treated with H_2_O_2_ showed an increased level of the DNA damage sensor histone *γ*-H2AX, displayed by immunoblotting and immunostaining already 1 and 6 hrs after treatment respectively (Fig. 1A and B). Using the comet assay, we could detect comet tails in H_2_O_2_-treated cells after 3 and 24 hrs (Fig. 1C), which is likely to represent single- or double-strand DNA breaks or apurinic and apyrimidinic sites.

In conclusion, the use of H_2_O_2_ is a suitable tool for inducing a broad range of DNA damage in the cancer cell line TE7, which is a prerequisite for the induction of desired molecular therapeutic consequences, such as cell cycle arrest or apoptosis.

### H_2_O_2_ treatment induces cell cycle arrest

**Fig. 2:**
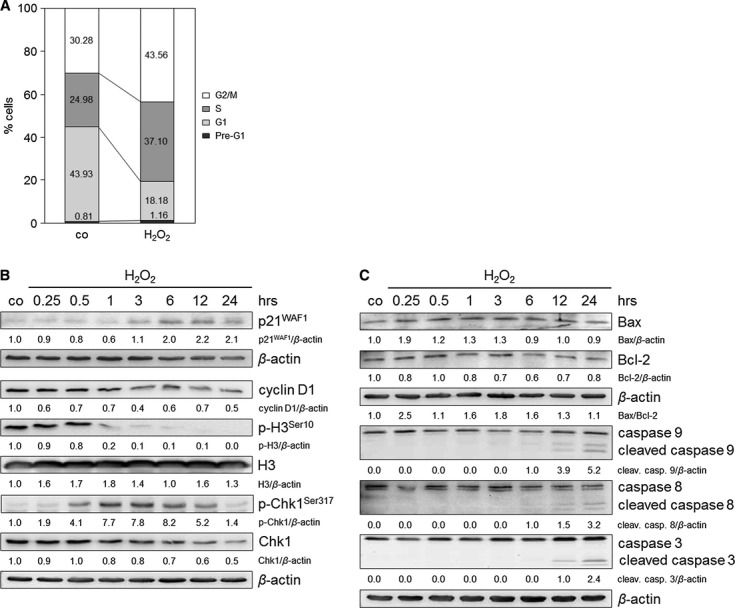
H_2_O_2_ induces cell cycle arrest and minor apoptosis in TE7 cells after treatment with 250 μM H_2_O_2_. (**A**) H_2_O_2_ induces S and G2 arrests 24 hrs after H_2_O_2_ treatment. Cell cycle profiles were determined by fluorescence-activated cell sorting (FACS) analysis, and the percentage of cells in the different cell cycle phases was calculated. The data are representative of three independent experiments. (**B**) H_2_O_2_ exposure induces an upregulation of p21^WAF1^, downregulation of cyclin D1 and p-H3^Ser10^ and activation of Chk1. Cells were cultured with H_2_O_2_ and grown for 0.25, 0.5, 1, 3, 6, 12 and 24 hrs. Lysates were immunoblotted and probed with anti-p21^WAF1^, -cyclin D1, -H3, -p-H3^Ser10^, -Chk1 and -p-Chk1^Ser317^ antibodies. *β*-actin was used as a loading control. Fold expression changes are given below the blots. (**C**) H_2_O_2_ initiates apoptosis pathways. Cells were incubated with H_2_O_2_ and lysates were immunoblotted for Bax, Bcl-2, caspases 8, 9 and 3 after 0.25, 0.5, 1, 3, 6, 12 and 24 hrs. The ratio of Bax/Bcl-2 is shown. *β*-actin was used as a loading control. Fold expression changes are given below the blots.

To prove whether H_2_O_2_-associated oxidative DNA damage induces growth inhibition effects in TE7 cells, cell cycle and cell proliferation analyses were performed. Indeed, we found 1.49- and 1.44-fold increased cell populations in the S and G2/M phases after 24 hrs respectively (Fig. 2A). As p21^WAF1^, a general cell cycle inhibitor, is able to block the cell cycle in both the G1/S and G2/M phases, its expression was analysed (Fig. 2B). The maximum of p21^WAF1^ expression was seen at 12 hrs after treatment. Moreover, we detected increased p21^WAF1^ mRNA expression, allowing us to conclude transcriptionally induced CDKN1A (Figure S1, Table S1). This justified our hypothesis that S and G2/M checkpoint arrests observed after 24 hrs were mediated by p21^WAF1^. We also found decreased expression of the G1 cell cycle regulator cyclin D1 and that of the late G2 and mitosis marker p-H3^Ser10^ from 1 to 24 hrs after H_2_O_2_ (Fig. 2B). This suggests that cell cycle arrest occurred in the S and early G2 phase, which is also supported by activating phosphorylation of Chk1 (Fig. 2B). Reduced BrdU incorporation reflected that H_2_O_2_-treated cells did not proliferate as strong as the control (Figure S2A).

### H_2_O_2_ induces minor apoptosis

Besides cell cycle arrest, DNA damage might also induce apoptosis. To determine possible H_2_O_2_-induced cell death and to distinguish apoptosis from necrosis, the Annexin-V assay was performed. To achieve maximal apoptosis and less necrosis, we elucidated the optimal H_2_O_2_ concentration, ranging from 10 to 500 μM (Figure S2B). A concentration of 250 μM H_2_O_2_ was sufficient not only to induce cell cycle arrest (Fig. 2A) but also to induce a more apoptotic than necrotic response (Figure S2B). Increase in the necrotic cell population was observed at 500 μM H_2_O_2_, reversing the ratio of apoptosis to necrosis. Apoptosis-regulating proteins, such as Bcl-2-associated X protein (Bax), caspases 9, 8 and 3, were slightly activated after 250 μM H_2_O_2_ (Fig. 2C). Notably, an increased Bax-to-B-cell CLL/lymphoma 2 (Bcl-2) ratio (Fig. 2C) joined with the cleavage of caspases 9 and 3 indicates the operation of the intrinsic pathway.

### Gene expression profile of H_2_O_2_-treated TE7 cells

**Fig. 3:**
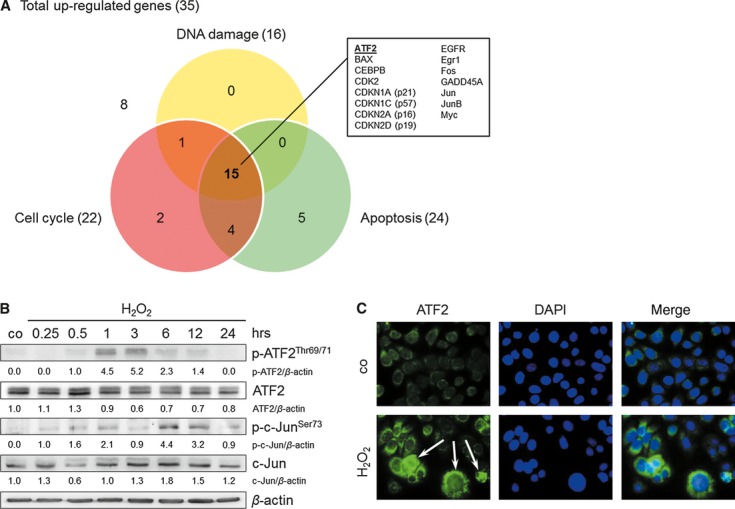
Identification of ATF2 as a key protein in the H_2_O_2_ response (250 μM) in TE7 cells. (**A**) Illustration of H_2_O_2_-upregulated genes involved in DNA damage, cell cycle and apoptosis regulation obtained using c-DNA microarray analyses. Upregulated ATF2 was the subject of further experiments. (**B**) ATF2 and c-Jun protein induction and activation by H_2_O_2_. Cells were treated with H_2_O_2_ and grown for 0.25, 0.5, 1, 3, 6, 12 and 24 hrs. Proteins were isolated and immunoblotted for ATF2, c-Jun and their activated forms p-ATF2^Thr69/71^ and p-c-Jun^Ser73^. *β*-actin was used as a loading control. Fold expression changes are given below the blots. (**C**) H_2_O_2_ induces cytoplasmic and nuclear accumulation of ATF2. Representative images of ATF2 immunostaining of H_2_O_2_-treated cells after 1 hr are shown. Nuclear DNA was visualized by 4′,6-diamidino-2-phenylindole (DAPI) counterstaining.

For the discovery of an oxidative stress-induced central key gene in TE7 cells regulating (*i*) DNA damage response, (*ii*) cell cycle progression and (*iii*) apoptosis, the mRNAs expression profile was determined using a cDNA microarray. Array analysis of H_2_O_2_-treated TE7 cells with the GEArray Q Series ‘Human Signal Transduction PathwayFinder Gene Array’ revealed various differentially activated genes belonging to several pathways (Figure S1, Table S1). We only obtained gene inductions, although 35 of 96 genes examined were upregulated (greater than 2.00-fold and newly expressed). These 35 genes were used for the creation of a Venn-diagram, focusing on their function in DNA damage, cell cycle progression and apoptosis (Fig. 3A). In summary, 15 of these 35 genes were found to be involved in all of these processes. Investigating these 15 genes, we were interested in selecting a transcription factor that has a putative function in oesophageal cancer, but that has not yet been described. Therefore, the database PubMed was used to prove whether the 15 genes play a role in oesophageal cancer (Table S2). Only two transcription factors, ATF2 and CCAAT/enhancer-binding protein beta (CEBPB), have not yet been described in oesophageal cancer. As the effectiveness of ATF2 is extended with a histone acetyltransferase (HAT) function, potentiating its transcription factor activity, we decided to target ATF2. Furthermore, novel studies led to the conclusion that ATF2 may play an important role in tumorigenesis (reviewed in [29]), suggesting ATF2 as a cancer drug target. As p21^WAF1^ might be ATF2-regulated [19, 22], we hypothesized that targeting ATF2 and therefore p21^WAF1^ is a promising strategy to switch from cell cycle arrest to enhanced apoptosis.

### ATF2 controls the expression of p21^WAF1^ and c-Jun

**Fig. 4:**
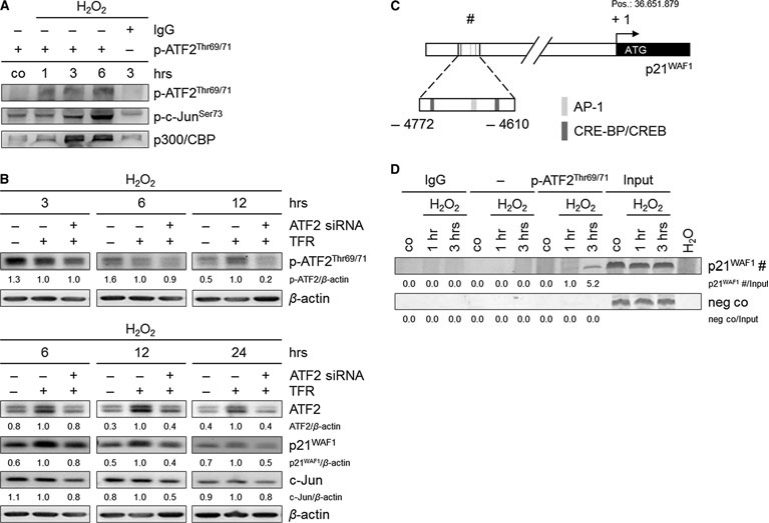
ATF2 regulates the expression of p21^WAF1^ and c-Jun, and p-ATF2^Thr69/71^ directly binds to the p21^WAF1^ promoter in H_2_O_2_-treated TE7 cells (250 μM). (**A**) p-ATF2^Thr69/71^ interacts with p-c-Jun^Ser73^ to form the AP-1 complex. In addition, p300 and CBP were found as p-ATF2^Thr69/71^ interaction partners. Cells subjected to H_2_O_2_ were lysed, and p-ATF2^Thr69/71^ was immunoprecipitated using anti-p-ATF2^Thr69/71^ antibody. Rabbit IgG was used as negative control. Precipitated lysates were immunoblotted for p-ATF2^Thr69/71^, p-c-Jun^Ser73^ and p300/CBP. (**B**) ATF2 knockdown causes a reduction in p-ATF2^Thr69/71^, ATF2, p21^WAF1^ and c-Jun protein expression. Cells were transfected with ATF2 siRNA and transfection reagent (TFR) for 7 hrs prior to H_2_O_2_ treatment. Thereafter, cells were grown for 3, 6, 12 and 24 hrs. Lysates were immunoblotted for p-ATF2^Thr69/71^, ATF2, p21^WAF1^ and c-Jun. *β*-actin was used as loading control. Fold expression changes are given below the blots. (**C**) Schematic illustration of the p21^WAF1^ promoter shows the putative ATF2-binding site (#), including the binding sequences AP-1, and CRE-BP/CREB −4772 to −4610 bp relative to the transcription start (+ 1; ATG, position 36.651.879), which was used for amplification. (**D**) p-ATF2^Thr69/71^ binds to the −4772 to −4610 bp p21^WAF1^ promoter region. Cells were treated with H_2_O_2_ and grown for 1 and 3 hrs. Cells were lysed, and p-ATF2^Thr69/71^ was immunoprecipitated using anti-p-ATF2^Thr69/71^ antibody. The target region (#) in the p21^WAF1^ promoter was analysed by semi-quantitative PCR. Rabbit IgG instead of anti-p-ATF2^Thr69/71^, an additional sample without antibody, and H_2_O served as negative controls. Fold PCR product accumulation is given below the gel photos.

ATF2, a component of the stress pathway, is activated *via* phosphorylation on threonine residues 69 and 71. It fulfils its transcriptional activity after complex formation as a homo- or heterodimer with p-c-Jun (AP-1 complex). Indeed, we found phosphorylation of ATF2, as well as of c-Jun already 30 and 15 min after H_2_O_2_ treatment respectively (Fig. 3B). ATF2 immunostaining revealed its cytoplasmic accumulation and, in a few cells, its slight nuclear accumulation after treatment (Fig. 3C). We analysed a complex formation between p-ATF2^Thr69/71^ and p-c-Jun^Ser73^ by co-immunoprecipitation that had revealed an interaction between both proteins upon treatment (Fig. 4A). This finding suggests that p-ATF2 may function as a heterodimer with p-c-Jun to form the AP-1 complex. Moreover, the HATs p300 and CREB-binding protein (CBP) were identified as interaction partners of p-ATF2^Thr69/71^ (Fig. 4A). This interaction might facilitate the accessibility of ATF2 itself and of other transcription factors to target gene promoters, such as the p21^WAF1^ promoter.

Next, we tried to answer the question of whether ATF2 may regulate the observed cell cycle arrest *via* p21^WAF1^, which is a prerequisite for the desired switch. Therefore, we performed ATF2 knockdown. The transfection of ATF2 siRNAs into the cells, which were subsequently exposed to H_2_O_2_, reduced the levels of activated p-ATF2^Thr69/71^ by about 10% and 80% at 6 and 12 hrs respectively (Fig. 4B). Indeed, we found decreased p21^WAF1^ expression of about 60% at 12 hrs and of about 50% at 24 hrs following ATF2 knockdown (Fig. 4B). This suggests that ATF2 may induce p21^WAF1^ expression as has been suggested. As the transfection procedure is equal to stress to the cells, it is not astonishing that proteins of the stress pathway, such as ATF2 and p21^WAF1^, were upregulated following addition of the TFR compared with untreated cells. As ATF2 knockdown also decreased c-Jun expression (Fig. 4B), we further suggest that ATF2 might positively regulate its own transactivation activity through an increased ATF2/c-Jun complex formation. In addition, following JNK inhibition and subsequent reduced ATF2 and c-Jun activation, we found suppressed p21^WAF1^ expression (Figure S3). This further supports that ATF2 is JNK-regulated, such as c-Jun, and controls p21^WAF1^ expression.

### ATF2 binds to a hitherto unknown ATF2-binding site in the p21^WAF1^ promoter

To prove whether ATF2 may directly regulate p21^WAF1^ expression at the transcriptional level, we aimed at detecting binding of p-ATF2^Thr69/71^ in the p21^WAF1^ promoter. We discovered an ATF2-binding site in the promoter sequence of the CDKN1A gene (NT_007592) taken from the National Center for Biotechnology Information (NCBI; http://www.ncbi.nlm.nih.gov/). The potential ATF2-binding site should span a high number of ATF2-binding sequences, such as CRE-BP, CREB, AP-1 and CRE-BP/CREB, within a short sequence area. Using the program TFSEARCH: Searching Transcriptions Factor Binding sites (Version 1.3, http://www.cbrc.jp/research/db/TFSEARCH.html), we found a potential binding site for ATF2, which is located at −4772 to −4610 bp far from the transcription start site (+ 1; ATG start codon at position 36.651.879). Moreover, this is an undescribed ATF2-binding site that includes two CRE-BP/CREB (TGAGGTCA/TGAGGTCA) and one AP-1 (GGTGACTCACT)-binding sequence (Fig. 4C). We analysed its potential p-ATF2^Thr69/71^-binding capacity by performing ChIP with the p-ATF2^Thr69/71^ antibody.

Indeed, ChIP experiments revealed the accumulation of the appropriate PCR product of the p21^WAF1^ promoter sequence 1 and 3 hrs after H_2_O_2_ treatment (Fig. 4D). In summary, we suggest a direct transcriptional induction of p21^WAF1^ through ATF2, presumably *via* the identified p-ATF2^Thr69/71^-binding site.

### ATF2 knockdown triggers the switch from cell cycle arrest to enhanced apoptosis

**Fig. 5:**
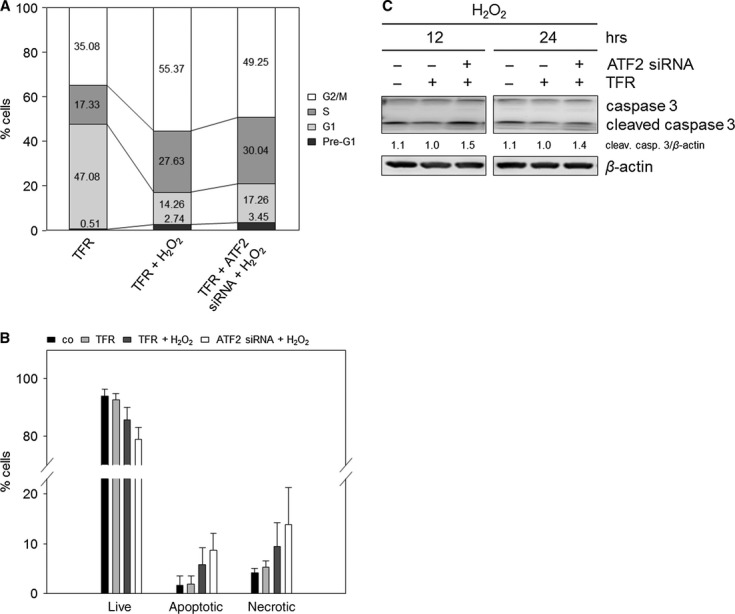
ATF2 knockdown combined with H_2_O_2_ induces a switch from cell cycle arrest to enhanced apoptosis in TE7 cells. Cells were transfected with ATF2 siRNA and transfection reagent (TFR) for 7 hrs prior to H_2_O_2_ treatment (250 μM). (**A**) H_2_O_2_ treatment combined with ATF2 knockdown reduces the G2/M arrest. After transfection and treatment, cells were grown for 24 hrs, and cell cycle analysis was performed. Differentially gated cell populations were counted; their percentage in the total cell populations was calculated and presented in the diagram. The data are representative of three independent experiments. (**B**) Combined treatment of H_2_O_2_ and ATF2 siRNA transfection reinforces apoptosis induction. Apoptotic, necrotic and live cell populations were measured after 24 hrs using Annexin-V and PI staining. Mean values are shown ± SD. (**C**) ATF2 knockdown induced elevated caspase 3 cleavage in H_2_O_2_-treated cells. The lysates were immunoblotted for caspase 3 after 12 and 24 hrs. *β*-actin was used as loading control. Fold expression changes are given below the blots.

To prove the hypothesis of switching cell cycle arrest to apoptosis by targeting the cell cycle arrest-inducing key protein, we performed ATF2 knockdown and analysed the induction of cell cycle arrest and apoptosis (Fig. 5). Cell cycle analyses clearly revealed a 28 % reduction in the G2/M arrest and an accumulation of S and G1 phase cell populations (Fig 5A). The decrease in G2/M arrest was accompanied by a 1.50-fold increase in apoptosis, indicated by the Annexin-V assay (Fig. 5B). This finding strongly supports a switch from cell cycle arrest to apoptosis as has been suggested. In addition, we also observed a 1.40-fold increase in the caspase 3 cleavage following ATF2 knockdown after 24 hrs (Fig. 5C). As p21^WAF1^ downregulation was observed following ATF2 knockdown (Fig. 4B), we concluded that p21^WAF1^ downregulation by targeting ATF2 induces the switch from cell cycle arrest to enhanced apoptosis. Casually, we observed a 1.47-fold increase in necrosis after ATF2 knockdown (Fig. 5B).

In summary, targeting of ATF2 caused a defect in G2/M checkpoint control with the potential to override the checkpoint, leading to increased apoptosis.

### ATF2-dependent G2/M arrest is a general feature of oxidative stress-exposed TE7 cells

**Fig. 6:**
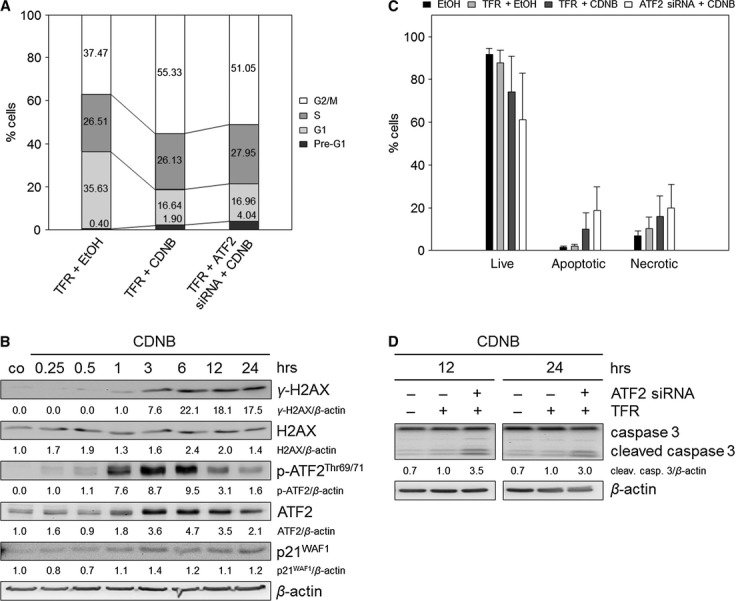
ATF2-mediated G2/M arrest as general feature following oxidative stress in TE7 cells. Reactive oxygen species production was evoked by the addition of 1-Chloro-2,4-dinitrobenzene (CDNB, 10 μM). Treatment with ethanol (EtOH) was used as control. (**A**) CDNB treatment combined with ATF2 knockdown reduces the G2/M arrest. Cells were transfected with ATF2 siRNA and transfection reagent (TFR) for 7 hrs prior to CDNB treatment. After transfection and treatment, cells were grown for 24 hrs, and cell cycle analysis was performed. Differentially gated cell populations were counted; their percentage in the total cell populations was calculated and presented in the diagram. The data are representative of three independent experiments. (**B**) CDNB induces *γ*-H2AX, p-ATF2^Thr69/71^ and p21^WAF1^. Cells were treated with CDNB and grown for 0.25, 0.5, 1, 3, 6, 12 and 24 hrs. Lysates were immunoblotted for *γ*-H2AX, H2AX, ATF2 and the activated form p-ATF2^Thr69/71^, as well as for p21^WAF1^. *β*-actin was used as loading control. Fold expression changes are given below the blots. (**C**) Combined treatment of CDNB and ATF2 siRNA transfection reinforces apoptosis induction. Apoptotic, necrotic and live cell populations were measured after 24 hrs using Annexin-V and PI staining. Mean values are shown ± SD. (**D**) ATF2 knockdown induced elevated caspase 3 cleavage in CDNB-treated cells. The lysates were immunoblotted for caspase 3 after 12 and 24 hrs. *β*-actin was used as loading control. Fold expression changes are given below the blots.

To assign the discovered molecular mechanisms not only to H_2_O_2_ but also to oxidative stress in general, we applied another oxidative stress-inducing tool, CDNB, which is known to elevate cellular ROS levels by reducing the anti-oxidative defence capacity [30, 31]. Indeed, CDNB treatment caused a 1.48-fold increase in cells in the G2/M phase (Fig. 6A), as well as nearly complete loss of BrdU incorporation (Figure S2A). Immunoblot analyses revealed induced ATF2 activation and p21^WAF1^ protein expression (Fig. 6B). Both are obviously associated with DNA damage, which is indicated by increased *γ*-H2AX (Fig. 6B). Furthermore, we could confirm that the CDNB-induced G2/M arrest was ATF2-dependent as indicated by a 24% reduction in G2/M arrest (Fig. 6A). Moreover, ATF2 knockdown caused a 1.87-fold increase in the apoptotic cell population after 24 hrs (Fig. 6C), also suggesting a switch from cell cycle arrest to apoptosis as shown for H_2_O_2_. Apoptosis induction after combined ATF2 siRNA and CDNB treatment was accompanied by increased caspase 3 cleavage after 24 hrs (Fig. 6D). Annexin-V assay revealed a 1.26-fold increase in necrosis following ATF2 knockdown (Fig. 6C). We also observed a slight increase in S and G1 cell population following AFT2 knockdown as shown for H_2_O_2_, suggesting that cells re-entered the cell cycle.

## Discussion

The understanding of the molecular mechanisms underlying oxidative stress response is the basis for improving anti-cancer therapies that have shown apoptosis resistance. Oxidative stress-induced DNA damage causes diverse responses in the cells. However, in the context of the aims of the therapies, we focused on cell cycle arrest. The aim was to overcome the observed cell cycle arrest and to switch cells to a greater extent to apoptosis by influencing a key protein.

Our data obtained with H_2_O_2_-treated TE7 cells reveal that ATF2 exerts a key role by partially mediating the G2/M arrest, thereby inhibiting apoptosis. In this process, ATF2 induced p21^WAF1^ presumably *via* transactivation. Thus, a combined treatment of oxidative stress with the knockdown of ATF2 caused better apoptotic effects in oesophageal cancer cells. ATF2 activation that mediated G2/M arrest was also observed in response to another pro-oxidant, thus suggesting a general response to oxidative stress.

### H_2_O_2_ induces DNA damage, cell cycle arrest, but minor apoptosis

H_2_O_2_-induced DNA damage led to apoptosis and cell cycle arrest. For the minor apoptosis observed, we concluded a participation of the intrinsic apoptotic pathway, by which elevated Bax expression led to the induction of caspase 9 and 3. In addition, we also observed caspase 8 activation, which may activate caspase 9 through BH3-interacting domain death agonist (Bid) [32]. Although caspases 8 and 9 were simultaneously activated, late activation of caspase 8 through caspase 9 cannot be excluded [33]. The low apoptotic events support the use of TE7 cells as a model for squamous oesophageal cancer, which exhibits only a poor apoptotic response to oxidative stress-based conventional radiochemotherapy [1]. Furthermore, the minor apoptosis observed may be a result of the fact that p53, as an apoptosis inductor, is transcriptionally repressed in TE-7 cells under non-stressed conditions, resulting in loss of detectable wild-type p53 protein even after DNA damage [34]. Moreover, as the examined cancer cell line also showed growth arrest, it can be used to study the switch from cell cycle arrest to reinforced apoptosis. We could demonstrate that an increase in p21^WAF1^ protein expression is caused by its transcriptional upregulation. Accordingly, p21^WAF1^ is able to establish the observed S and G2 arrests. Thus, on the basis of these results, we propose a molecular underlying mechanism that inhibits apoptosis by growth arrest.

### Identification of ATF2 as a potential target to increase apoptosis sensitivity

Targeting DNA damage checkpoint proteins, such as Chk1, is a straightforward strategy in anti-cancer therapy (reviewed in [17]). We therefore aimed at identifying a potential DNA damage checkpoint protein. The potential protein should display a cell cycle regulator by itself, or should regulate a cell cycle regulator as transcription factor, switching to reinforced apoptosis sensitivity. Array analyses gave rise to the transcriptional activation of ATF2, a transcription factor that may affect many genes, including genes regulating cell cycle progression ([35–37], reviewed in [38]), such as p21^WAF1^ [19, 22] and growth arrest and DNA damage-inducible protein 45 (GADD45) [39], as well as the anti-apoptotic Bcl-2 [40]. Indeed, Ronai *et al*. reported a hypersensitization of melanoma to irradiation after inactivation of ATF2 [41]. Moreover, Bhoumik *et al*. pointed out that an ATF2 peptide derived from amino acids 51 to 100 of ATF2 is useful to overcome apoptosis resistance in melanoma [42, 43]. Thus, the authors highlight the possible use of the ATF2-derived peptides in drug design, suggesting its feasibility in clinical practice. Importantly, the xenograft model in nude mice, subcutaneously injected with LU1205 or FEMX human melanoma tumour-derived cell lines that constitutively express ATF2 (51-100) peptide, showed inhibition of human melanoma cell growth *in vivo* [44].

### ATF2 mediates G2/M cell cycle arrest, thereby inhibiting apoptosis

ATF2 knockdown experiments demonstrated that ATF2 mediates in part the establishment of the G2/M checkpoint arrest. In this context, we identified p21^WAF1^ as an ATF2 target. Furthermore, inhibition of JNK activity also decreased p21^WAF1^ expression following less activation of ATF2, suggesting that JNK is an upstream activator of ATF2 [45] and p21^WAF1^ is ATF2-regulated. Combined CDNB and ATF2 siRNA treatment revealed that ATF2-dependent G2/M arrest may be a general feature caused by oxidative stress response. Thus, targeting of ATF2 should cause defective G2/M checkpoint control with the potential to override the checkpoint leading to increased apoptosis [46]. Indeed, we found reinforced apoptosis following ATF2 knockdown as a consequence of defective G2/M checkpoint. The reversed apoptosis-to-necrosis ratios in the knockdown experiments, especially after H_2_O_2_ or CDNB treatment, are addressed to the transfection medium.

### Identification of a novel ATF2-binding site in the p21^WAF1^ promoter

ATF2 knockdown clearly revealed p21^WAF1^ as a target of ATF2 in TE7 cells. In the p21^WAF1^ promoter, one binding site upstream of the transcription start at the position 36.651.879 for ATF2 has been reported [19]. This known binding site is located between the −6877 and −5365 bp promoter region relative to the transcriptional initiation site. Here, we identified region −4772 to −4610 bp relative to position 36.651.879 as a novel ATF2-binding site. This sequence contains two CRE-BP/CREB (TGAGGTCA/TGAGGTCA) and one AP-1 site (GGTGACTCACT) within a short sequence area. As a consequence, ATF2 should be able to bind as a homodimer at CRE-BP/CREB sequence or as a heterodimer at the AP-1 site. However, additional binding of ATF2 to other p21^WAF1^ promoter sites cannot be excluded. Moreover, we have shown that p21^WAF1^ gene induction may also be regulated by other transcription factors, such as tumour protein p53 [25].

In addition, we could show that c-Jun expression was regulated by ATF2, as shown by Fu *et al*. [47]. This could be the driving force for transcriptional activity of ATF2 with regard to heterodimerization to form AP-1 also in our model [48]. In contrast, ATF2 inhibition in melanoma cells increased c-Jun expression [42, 49].

### Proposed model: ATF2 knockdown combined with oxidative stress switches from cell cycle arrest to reinforced apoptosis

**Fig. 7:**
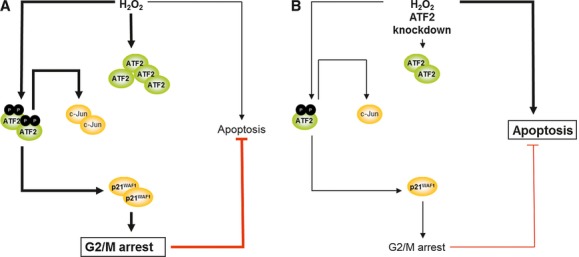
Mechanistic model of the TE7 cell response to H_2_O_2_ and to H_2_O_2_ plus ATF2 knockdown. (**A**) H_2_O_2_ exposure causes DNA damage and consequently the activation of ATF2 *via* phosphorylation on threonine residues 69 and 71. This transcription factor induces the transcription of p21^WAF1^ and c-Jun. As ATF2 induces the G2/M growth arrest *via* p21^WAF1^, ATF2 thereby inhibits apoptosis. (**B**) The combination of H_2_O_2_ stress with the knockdown of ATF2 leads to reduced ATF2 protein level, causing a lesser amount of c-Jun and, most notably, of p21^WAF1^. Consequently, G2/M arrest is reduced, but apoptosis reinforced.

The results presented here may answer the question of how an ATF2 inhibition can increase apoptosis sensitivity (Fig. 7). H_2_O_2_-mediated oxidative stress causes both induction and phosphorylation of ATF2, thereby activating this transcription factor (Fig. 7A). Then, p-ATF2^Thr69/71^ induces the transcription of the cell cycle inhibitor p21^WAF1^ and that of c-Jun. In addition, ATF2 partially induces the G2/M growth arrest *via* p21^WAF1^ induction, thereby inhibiting the apoptotic cell death.

Indeed, the combination of oxidative stress treatment with the knockdown of ATF2 (Fig. 7B) led to reduced p21^WAF1^ protein amount and, thereby enhanced apoptosis. Importantly, we recently observed that p21^WAF1^ downregulation also reinforced apoptosis sensitivity of colorectal cancer cells [25]. This finding further supports the importance of p21^WAF1^ in (*i* ) the establishment of cell cycle arrest, (*ii* ) apoptosis inhibition and therefore (*iii* ), in our model, in switching cell cycle arrest to apoptosis. The reduction in p21^WAF1^ expression, in turn, caused reduced G2/M arrest, and thereby increased apoptosis. Consequently, we suggest that decreased G2/M checkpoint activation as a result of decreased ATF2 levels may be responsible for apoptosis induction: cells with damaged DNA override the G2/M checkpoint *via* mitotic slippage, which finally leads to mitotic catastrophe and cell cycle re-entry [26]. In support of this, we observed both increased apoptosis, as well as increased G1 and S phase cell population.

We could recently show that targeting the promoter region of p21^WAF1^ is a promising strategy to switch from cell cycle arrest to enhanced apoptosis [25]. Therefore, targeting p21^WAF1^ by ATF2 inhibition supports our previous data. Moreover, ATF2 possesses an intrinsic HAT activity [50], conferring ATF2 an epigenetic activity. Thus, ATF2 inhibition could prevent both its operation as a transcription factor and a change in the histone acetylation status around promoter regions. To the best of our knowledge, we are the first to show the mechanism of elevating apoptosis through ATF2 knockdown under oxidative stress in squamous oesophageal cancer cells. This may emphasize the important role of ATF2 in DNA damage checkpoint control in cancer.

## Supporting information

**Figure S1.** H_2_O_2_ causes transcriptional upregulation of genes of diverse signal transduction pathways in TE7 cells. Differential gene expression profile was detected by cDNA microarray analysis. Cells were treated with 250 μM H_2_O_2_ or were left untreated (co) and grown for 30 min. Subsequently, the RNA was isolated and reverse transcription was performed. The cDNA was hybridized with the fixed cDNA samples of the ‘GEArray Q Series Human Signal Transduction PathwayFinder Gene Array’. The hybridization extent was measured by chemiluminescence and evaluated by densitometry. Blank value was subtracted from gene raw values. The received gene values were adjusted with the housekeeping gene *β*-actin value by calculating the ratio.

**Figure S2.** H_2_O_2_ decreases proliferation and increases apoptosis. (A) 5-bromo-2′-deoxyuridine (BrdU) proliferation ELISA showed reduced proliferation of H_2_O_2_-treated TE7 cells, as well as abolished proliferation of CDNB-treated TE7 cells. Cells were treated with 250 μM H_2_O_2_ or 10 μM CDNB or were left untreated or treated with ethanol, and grown for 24 hrs. BrdU assay was performed as described in Materials and methods by measuring the absorbance of BrdU-positive cells at 450 nm following treatment. Data are mean ± SD. (B) H_2_O_2_ causes apoptosis and necrosis in a dose-dependent manner. Apoptotic, necrotic and live cell populations were measured after 24 hrs using Annexin-V and PI staining. Mean values are shown ± SD.

**Figure S3.** p21^WAF1^ is induced *via* the JNK-ATF2-pathway after H_2_O_2_ treatment (250 μM) in TE7 cells. Inhibition of JNK activity using SP600125 revealed decreased activation of ATF2 and c-Jun, therefore causing reduced p21^WAF1^ expression. Cells were treated with H_2_O_2_ and grown for 1, 3, 6, 12, and 24 hrs following pre-incubation for 1 hr with 10 μM of JNK inhibitor SP600125. Lysates were immunoblotted for total ATF2 and c-Jun, as well as for their activated forms p-ATF2^Thr69/71^ and p-c-Jun^Ser73^, and for p21^WAF1^. *β*-actin was used as loading control. Fold expression changes are given below the blots.

**Table S1.** Differential gene expression profile of H_2_O_2_-treated TE7 cells detected by cDNA microarray analysis (Figure S1).

**Table S2** PubMed search for potential target genes in oesophageal cancer. State 01/30/13.
