# Supplementary figures and images for: ATF2 knockdown reinforces oxidative stress-induced apoptosis in TE7 cancer cells
Source: J Cell Mol Med. 2013 Jun 25;17(8):976–88. doi: 10.1111/jcmm.12071 (PMC3780530; doi:10.1111/jcmm.12071)

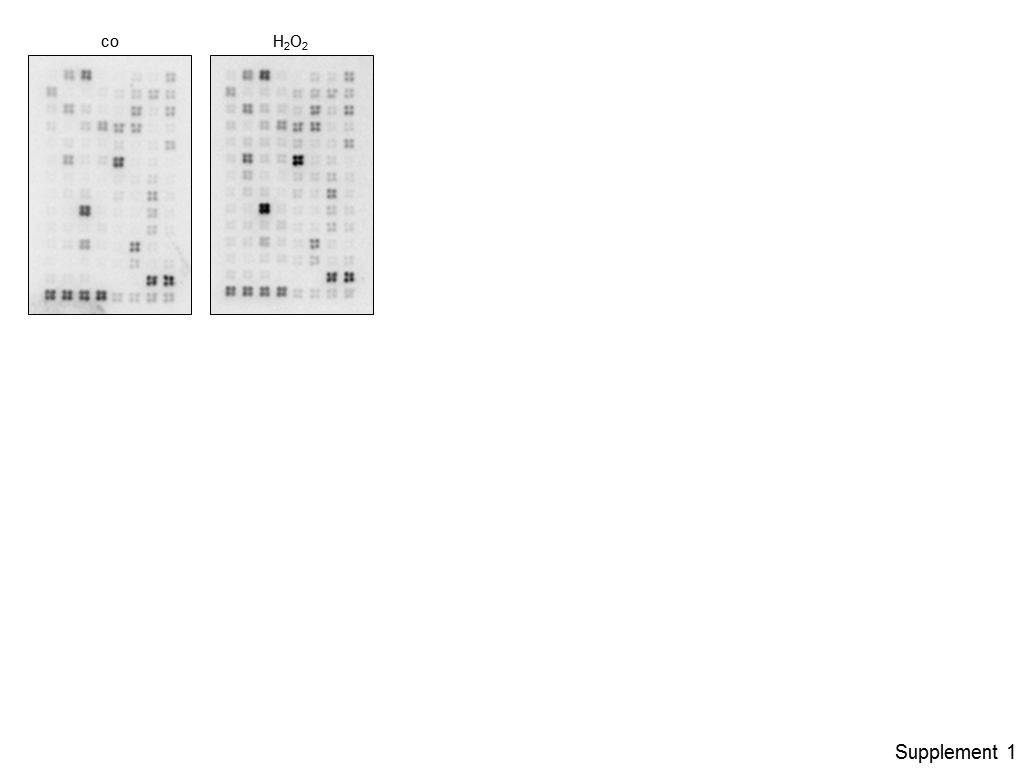

Supplement: Supplementary file 1 [file jcmm0017-0976-SD1.tif]

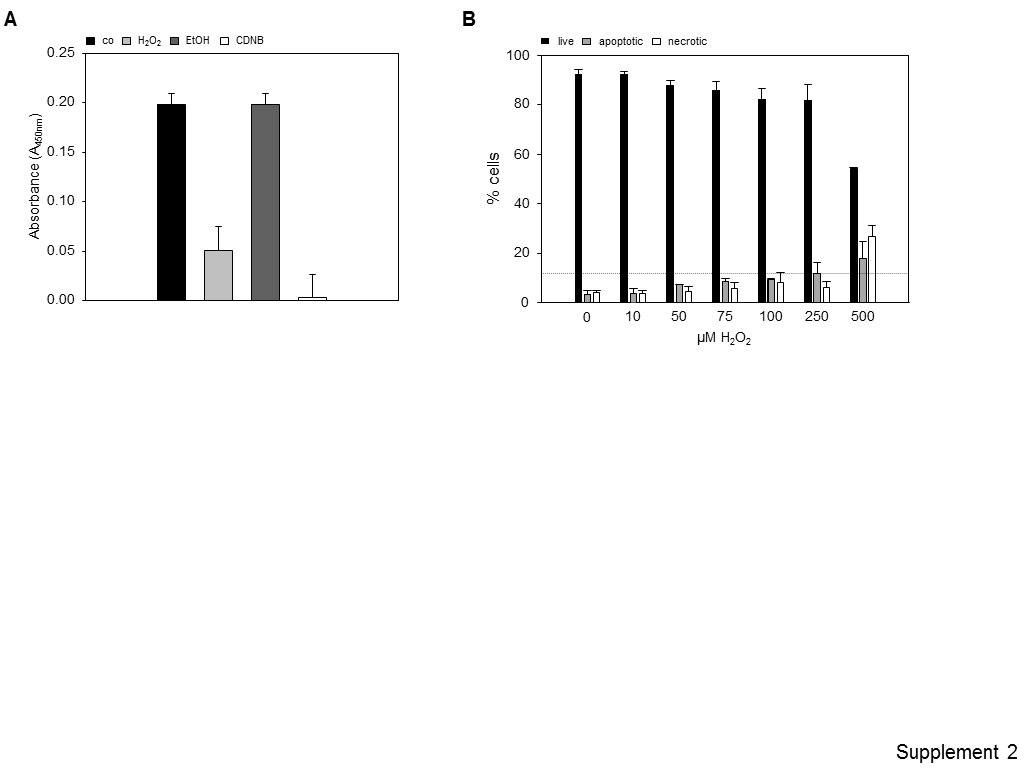

Supplement: Supplementary file 2 [file jcmm0017-0976-SD2.tif]

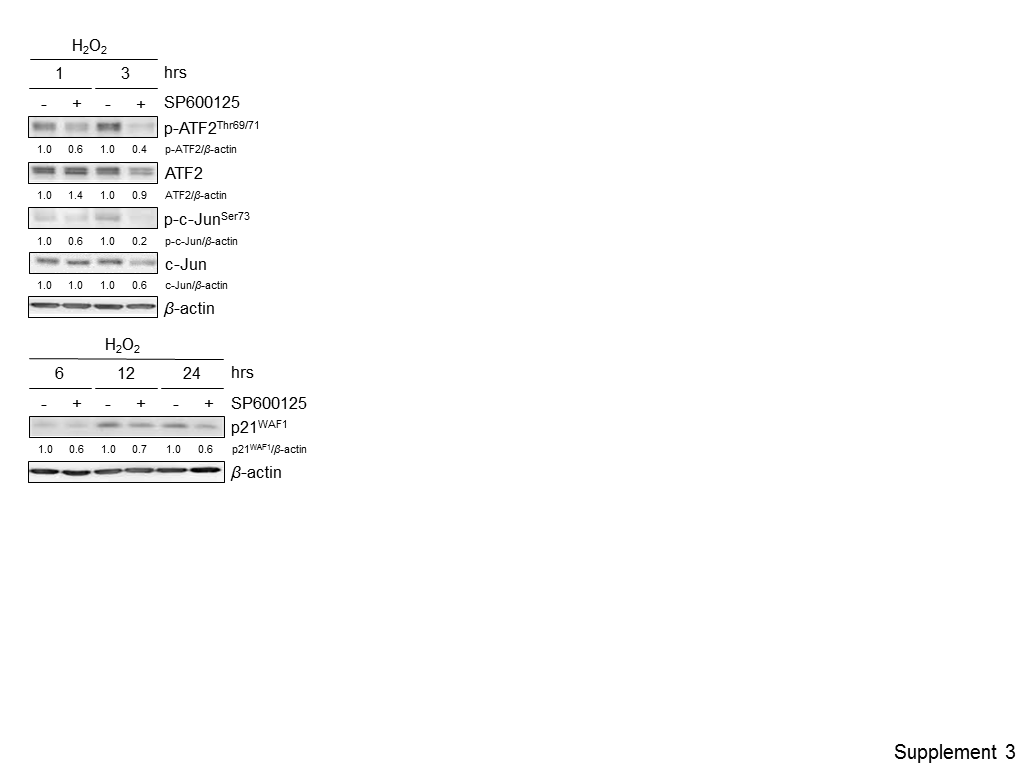

Supplement: Supplementary file 3 [file jcmm0017-0976-SD3.tif]
