# Supplementary material for: ATF2 knockdown reinforces oxidative stress-induced apoptosis in TE7 cancer cells
Source: J Cell Mol Med. 2013 Jun 25;17(8):976–88. doi: 10.1111/jcmm.12071 (PMC3780530; doi:10.1111/jcmm.12071)
Supplement: Supplementary file 4 [file jcmm0017-0976-SD4.docx]

### Tables

**Table S1** Differential gene expression profile of H_2_O_2_-treated TE7 cells detected by cDNA microarray analysis (Fig S1).

| **Pathway** | **Gene** | **Fold change** | **GenBank** |
| --- | --- | --- | --- |
| **Mitogenic Pathway** | | | |
|  | | | |
| EGR1 | Early growth response 1 | 2.96 ↑ | X52541 |
| FOS | Human cellular oncogene c-fos | ne | V01512 |
| JUN | v-jun avian sarcoma virus 17 oncogene homolog | ne | J04111 |
| **Wnt Pathway** | | | |
|  | | | |
| CCND1 | Cyclin D1 (PRAD1: parathyroid adenomatosis 1) | ne | M64349 |
| JUN | v-jun avian sarcoma virus 17 oncogene homolog | ne | J04111 |
| MYC | v-myc avian myelocytomatosis viral oncogene homolog | 2.41 ↑ | X00364 |
| **Hedgehog Pathway** | | | |
|  | | | |
| HIP | Hedgehog-interacting protein | ne | AY009317 |
| HNF3B | Hepatocyte nuclear factor 3, beta | 3.22 ↑ | AB028021 |
| PTCH2 | Patched (Drosophila) homolog 2 | 4.86 ↑ | AF091501 |
| **TGF-ß Pathway** | | | |
|  | | | |
| CDKN1A | Cyclin-dependent kinase inhibitor 1A (p21^WAF1^) | 3.76 ↑ | L47233 |
| CDKN1C | Cyclin-dependent kinase inhibitor 1C (p57, Kip2) | 2.77 ↑ | U22398 |
| CDKN2A | Cyclin-dependent kinase inhibitor 2A (melanoma, p16, inhibits CDK4) | 5.16 ↑ | U26727 |
| CDKN2B | Cyclin-dependent kinase inhibitor 2B (p15, inhibits CDK4) | ne | L36844 |
| CDKN2C | Cyclin-dependent kinase inhibitor 2C (p18, inhibits CDK4) | 22.72 ↑ | U17074 |
| CDKN2D | Cyclin-dependent kinase inhibitor 2D (p19, inhibits CDK4) | 2.24 ↑ | U40343 |
| **Survival Pathway-PI3 Kinase/AKT Pathway** | | | |
|  | | | |
| CCND1 | Cyclin D1 (PRAD1: parathyroid adenomatosis 1) | ne | M64349 |
| JUN | v-jun avian sarcoma virus 17 oncogene homolog | ne | J04111 |
| MYC | v-myc avian myelocytomatosis viral oncogene homolog | 2.41 ↑ | X00364 |
| **Survival Pathway- NF-κB Pathway** | | | |
|  | | | |
| BIRC2 | Homo sapiens baculoviral IAP repeat-containing 2 | 3.85 ↑ | U45879 |
| BIRC3 | Inhibitor of apoptosis protein 1 | 2.27 ↑ | U37546 |
| **p53 Pathway** | | | |
|  | | | |
| BAX | BCL2-associated X protein | 2.23 ↑ | L22474 |
| CDKN1A | Cyclin-dependent kinase inhibitor 1A (p21Cip1) | 3.76 ↑ | L47233 |
| GADD45A | DNA-damage-inducible transcript 1 | ne | M60974 |
| **Stress Pathway** | | | |
|  | | | |
| ATF2 | Activating transcription factor 2 | 2.68 ↑ | X15875 |
| FOS | Human cellular oncogene c-fos | ne | V01512 |
| HSPB1 | Heat shock 27 KD protein | 3.65 ↑ | Z23090 |
| HSPCA | Hsp90 (Human mRNA for 90-kDa heat-shock protein) | 2.39 ↑ | X15183 |
| MYC | v-myc avian myelocytomatosis viral oncogene homolog | 2.41 ↑ | X00364 |
| **NFκB Pathway** | | | |
|  | | | |
| ICAM1 | Intercellular adhesion molecule 1 (CD54), human rhinovirus receptor | ne | NM_000201 |
| **NFAT Pathway** | | | |
|  | | | |
| CD5 | T-cell surface glycoprotein CD5 | 3 | X04391 |
| **CREB Pathway** | | | |
|  | | | |
| CYP19 | Cytochrome P450, subfamily XIX (aromatization of androgens) | 2.17 ↑ | Y07508 |
| EGR1 | Early growth response 1 | 2.96 ↑ | X52541 |
| FOS | Human cellular oncogene c-fos | ne | V01512 |
| **Jak-Stat Pathway** | | | |
|  | | | |
| IL4 | Interleukin 4 | ne | M13982 |
| **Estrogen Pathway** | | | |
|  | | | |

| CTSD | Cathepsin D (lysosomal aspartyl protease) | 3.49 ↑ | M11233 |
| --- | --- | --- | --- |

| EGFR | Epidermal growth factor receptor | 2.33 ↑ | X00588 |
| --- | --- | --- | --- |
| **Androgen Pathway** | | | |
|  | | | |
| CDK2 | Cyclin-dependent kinase 2 | 2.47 ↑ | X61622 |
| CDKN1A | Cyclin-dependent kinase inhibitor 1A (p21Cip1) | 3.76 ↑ | L47233 |
| EGFR | Epidermal growth factor receptor | 2.33 ↑ | X00588 |
| KLK2 | Prostrate kallikrein 2 | ne | NM_005551 |
| **Calcium and Protein Kinase C Pathway** | | | |
|  | | | |
| FOS | Human cellular oncogene c-fos | ne | V01512 |
| JUN | v-jun avian sarcoma virus 17 oncogene homolog | ne | J04111 |
| MYC | v-myc avian myelocytomatosis viral oncogene homolog | 2.41 ↑ | X00364 |
| ODC1 | Human omithine decarboxylase gene | 2.23 ↑ | M16650 |
| **Phospholipase C Pathway** | | | |
|  | | | |
| EGR1 | Early growth response 1 | 2.96 ↑ | X52541 |
| FOS | Human cellular oncogene c-fos | ne | V01512 |
| ICAM1 | Intercellular adhesion molecule 1 (CD54), human rhinovirus receptor | ne | NM_000201 |
| JUN | v-jun avian sarcoma virus 17 oncogene homolog | ne | J04111 |
| JUNB | Jun-B (Jun B proto-oncogene) | ne | X51345 |
| **Insulin Pathway** | | | |
|  | | | |
| CEBPB | Homo sapiens CCAAT/enhancer binding protein (C/EBP), beta | 3.24 ↑ | NM_005194 |
| GYS1 | Homo sapiens glycogen synthase 1 (muscle) | 4.73 ↑ | NM_002103 |
| **Retinoic Acid Pathway** | | | |
|  | | | |
| CDX1 | Homo sapiens caudal type homeo box transcription factor 1 | ne | NM_001804 |

| CTSD | Cathepsin D (lysosomal aspartyl protease) | 3.49 ↑ | M11233 |
| --- | --- | --- | --- |

| HOXB1 | Homo sapiens homeo box B1 | 3.34 ↑ | NM_002144 |
| --- | --- | --- | --- |

^ne^ indicates newly expressed genes following H_2_O_2_ treatment, with a low basal background expression under normal growth condition. ^↑^ displays an upregulation of the appropriate gene.

**Table S2** PubMed search for potential target genes in esophageal cancer. State 01/30/13.

| Gene/protein | esophagus cancer | esophageal cancer |
| --- | --- | --- |
| ATF2 | 0 | 0 |
| BAX | 90 | 96 |
| CEBPB | 0 | 0 |
| CDK2 | 15 | 15 |
| CDKN1A (p21) | 167 | 168 |
| CDKN1C (p57) | 6 | 6 |
| CDKN2A (p16) | 246 | 239 |
| CDKN2D (p19) | 1 | 1 |
| EGFR | 223 | 237 |
| Egr1 | 11 | 11 |
| Fos | 18 | 16 |
| GADD45A | 8 | 8 |
| Jun | 38 | 48 |
| JunB | 2 | 2 |
| Myc | 139 | 152 |
