# Supplementary material for: ATF2 knockdown reinforces oxidative stress-induced apoptosis in TE7 cancer cells
Source: J Cell Mol Med. 2013 Jun 25;17(8):976–88. doi: 10.1111/jcmm.12071 (PMC3780530; doi:10.1111/jcmm.12071)
Supplement: Supplementary file 5 [file jcmm0017-0976-SD5.doc]

**Supporting Information**

**Material and Methods**

**cDNA array analysis.**

Total RNA was prepared using the TRIzol reagent (Life Technologies GmbH, Darmstadt, Germany), and 3 µg of RNA was used for reverse transcription. The probe was labeled with biotin-16-2-deoxyuridine-5´-triphosphated (biotin-16-dUTP) using the Ampolabeling LRP Kit of SuperArray Bioscience Corporation according to the manufacturer’s instructions. Microarray hybridization was performed according to the manufacturer’s protocol. Images were processed, evaluated, and normalized. Normalized, corrected signal values of the genes were used to calculate the relative expression by building gene/housekeeping gene ratio multiplied by 100. x-fold expression of a gene after H_2_O_2_ treatment in comparison to the control was obtained by generating the ratio of the relative expression of a gene after H_2_O_2_ treatment to the relative expression of the gene of the control sample. The genes that showed marked differences after H_2_O_2_ treatment were listed based on an at least 2-fold change of expression and a gene/housekeeping gene ratio multiplied by 100 of the control sample greater than 20. Newly expressed genes following H_2_O_2_ treatment are defined by a gene/housekeeping gene ratio multiplied by 100 of the control sample less than 20 and by a gene/housekeeping gene ratio multiplied by 100 of the H_2_O_2_-treated sample greater than 100.

# Bioinformatic databases

We chose a sequence - 4772 to - 4610 bp far of the transcription start (position 36.651.879) of the CDKN1A gene, including two CRE-BP/CREB and one AP-1 sequence. To design primers for the Chromatin immunoprecipitation experiments, the program “Primo Find 3.4” from <http://www.changbioscience.com/primo/primof.html> was used. To address protein functions, we used the following data bases: Database for Annotation, Visualization and Integrated Discovery (DAVID, [http://david.abcc.ncifcrf.gov](http://david.abcc.ncifcrf.gov/)) and UniProt Knowledgebase (UniProtKB, [www.uniprot.org](http://www.uniprot.org/)).

# Chromatin immunoprecipitation assay (ChIP)

Crosslinking was accomplished with methanol-free formaldehyde. Lysis, shearing buffers, and ChIP buffer 1 and 2 were additionally supplemented with phosphatase inhibitor (1 : 20, Active Motif) to assure sustaining of ATF2 phosphorylation. Extracts containing the chromatin were sonicated, leading to the formation of DNA fragments with an average size of 500 bp. The resulting solution was cleared by centrifugation 12 min at maximum speed at 4 °C. Chromatin was pre-cleared with magnetic beads for 1 hr at 4 °C. The protein concentration of the extract was measured, and 500 µg of protein was used for the immunoprecipitation reaction at 4 °C overnight with 2 µg of anti-p-ATF2^Thr69/71^ antibody (Cell Signaling Technology, Inc.). Negative ChIP controls were 500 µg of protein added with 2 µg of normal rabbit immunoglobulin G (IgG, Cell Signaling Technology, Inc.) or without antibody. p21^WAF1^ promoter primers were sense 5’-TTACGCCACTGTGTCCACC-3’ and antisense 5’- CGGGGTTCTGACCTCATAC-3’. Negative control primers were sense 5’-TGT TCCGCCTCTTCTCTGG-3’ and antisense 5’-AAGGGAGAGGAAGTGCTGG-3’. PCR products were unraveled on 8 % polyacrylamide gels by electrophoresis. Finally, gels were silver-stained. PCR amplification of the immunoprecipitated DNA was normalized to the appropriate input DNA sample amount.

# Co-Immunoprecipitation

Whole cell lysates were briefly sonicated and pre-cleared at 4 °C for 1 hr using protein G-magnetic beads (Active Motif). Cell lysates (250 µg proteins) were incubated with 40 ng p‑ATF-2^Thr69/71^ or 40 ng normal rabbit IgG (Cell Signaling Technology, Inc.) overnight at 4 °C, and afterwards, protein G-magnetic beads were added for 3 hrs at 4 °C. The beads were washed six times, and subsequently heated at 95 °C for 5 min. Proteins were separated by SDS-polyacrylamide gel electrophoresis, followed by immunoblotting.
